# Job Satisfaction and Burnout in Croatian Physiotherapists

**DOI:** 10.3390/healthcare10050905

**Published:** 2022-05-13

**Authors:** Patricija Puhanić, Suzana Erić, Jasminka Talapko, Ivana Škrlec

**Affiliations:** 1 Faculty of Dental Medicine and Health, Josip Juraj Strossmayer University of Osijek, 31000 Osijek, Croatia; ppuhanic24@gmail.com (P.P.); jtalapko@fdmz.hr (J.T.); 2 Department of Radiotherapy and Oncology, Clinical Hospital Center Osijek, 31000 Osijek, Croatia; eric.suzana@kbo.hr; 3 School of Medicine, Josip Juraj Strossmayer University of Osijek, 31000 Osijek, Croatia

**Keywords:** burnout, disengagement, exhaustion, job satisfaction, physiotherapists

## Abstract

Background: Physiotherapists are important healthcare professionals in modern and multidisciplinary health forces. However, they are exposed to a high risk of occupational burnout, which is associated with reduced job satisfaction. Job satisfaction is essential for medical professionals because it directly influences patient safety and the quality of medical care. Therefore, this study aimed to determine the association between sociodemographic variables of Croatian physiotherapists, job satisfaction, and occupational burnout. Methods: A cross-sectional study was performed among 404 physiotherapists using a sociodemographic questionnaire, the Job Descriptive Index (JDI), and Oldenburg Burnout Inventory (OLBI). Results: The study group of Croatian physiotherapists was marked by a high level of job satisfaction and occupational burnout. However, a higher level of occupational burnout is associated with lower job satisfaction. The main determinants of job satisfaction were younger age, female gender, less work experience, and married or partnership. At the same time, a higher level of occupational burnout was associated with working in government institutions and being single. Conclusions: As a reaction to psychological stress at work with the main components of exhaustion and disengagement, occupational burnout is negatively associated with job satisfaction. Therefore, assessing the factors influencing job satisfaction and burnout in the workplace can help develop physiotherapists’ mental health prevention strategies.

## 1. Introduction

Physiotherapists are unique and essential healthcare professionals in modern, multidisciplinary health forces, contributing to the health economy in acute care and rehabilitation settings, primary care, prevention, and public health [1,2]. Due to the nature of work, physiotherapists are exposed to a high risk of burnout, which is associated with reduced job satisfaction and mistakes in the workplace [3].

Job satisfaction is determined by an individual’s attitude towards a job and depends on their emotional experiences at work [4]. It is most often defined as an individual’s pleasant or positive emotional state that arises from realizing their expectations and needs at work [5]. Everyday events in the workplace will affect the employee’s level of satisfaction. Still, these events will not significantly change the employee’s overall feelings towards the organization in which they work. Therefore, job satisfaction is an individual’s response to specific aspects of their work [6]. Job satisfaction is affected by various factors [7]. Intrinsic factors, such as the work environment, are the dominant predictive factors for physiotherapists’ job satisfaction [8]. Extrinsic factors, such as salary, promotion, and the opportunity for professional development, have a lesser effect on job satisfaction [9]. Job satisfaction among healthcare professionals is very important as it directly affects the patient safety and quality of healthcare services [5]. Decreased job satisfaction is common in many healthcare professions and can significantly affect hiring and retaining employees. Reduced job satisfaction can lead to a reduced quality of patient care and can manifest as burnout, anxiety, and depression [10]. In addition, reduced job satisfaction can affect absenteeism or resignation. Job satisfaction is associated with performance at work, hope, optimism, and resilience, showing a direct link between job satisfaction and the psychological state. In addition, job satisfaction positively impacts patients and the work environment. Pay levels and communication within the team also affect job satisfaction [11]. Career insight and job satisfaction can help keep qualified physiotherapists in active practice and inform policymakers in workforce planning [12]. Health systems can only work with healthcare professionals. Therefore, healthcare workers must be equitably distributed and accessible to the population. Moreover, they must possess the necessary competencies and be motivated to provide quality care that is appropriate and acceptable [13]. This is particularly important in the economy after COVID-19, as many countries face challenges in financing the health sector. Therefore, it is essential to emphasize the need for adequate health workforce planning to achieve optimal productivity and performance [13].

Burnout is a set of psychological symptoms that result from prolonged exposure to stressors [9,14]. Occupational burnout is a state of mental and physical exhaustion due to the experience of failure and excessive social and physical demands at work. Numerous factors could lead to burnout. Job demands and resources are one of them [15]. Job demands include the workload, role ambiguity, role conflict, stress, stressful events, work pressure, lack of social support, autonomy and skill [16]. An imbalance between job demands and resources creates stress, leading to occupational burnout [15]. Additionally, stressors could be associated with individual personality, and it is challenging to mention all possible factors that could cause burnout. However, the personal resources of employees could help them cope with job demands, such as optimism, self-efficacy, and resilience [16]. Healthcare professionals have a high burnout rate due to their involvement in patient care [17], and several studies have shown that burnout rates among healthcare professionals are increasing [5,18]. The prevalence of burnout syndrome among medical staff ranges from 10% to 80.5% [19]. Previous studies have identified a high prevalence of burnout in the medical profession [20,21,22]. Physical therapy performed on patients with severely impaired physical health is very stressful and requires a high level of responsibility and involvement of physiotherapists. All this can affect the mental state and health of the physiotherapist [5,15,20,23]. Physiotherapists provide emotional and physical support to patients, often facing various conditions of disability and aggressive and depressive behavior of patients [18]. In addition to being exposed to stressors at work and low-to-moderate levels of burnout, physiotherapists are also exposed to high workloads, musculoskeletal disorders, various infections, and physically demanding interventions that can cause diverse spinal diseases [3,9,14,24]. For these reasons, physiotherapists are exposed to high-stress levels at work and an increased risk of occupational burnout [24,25,26]. Occupational burnout can lead to drug and alcohol abuse and depression, which significantly affects the quality of medical care and treatment [23,26,27]. In addition, it is associated with an increased number of suicides [21].

As the job of a physiotherapist can be very stressful, a heavy workload creates stress that can result in poor healthcare [8]. Stress at work leads to burnout, reducing physiotherapists’ ability to meet work requirements [5]. Job satisfaction is a protective factor against burnout and the negative consequences of stress at work [5]. Physiotherapist burnout can cause increased medical errors and decreased job satisfaction [21].

However, due to the current pandemic of the disease COVID-19, medical professionals are affected because they are under enormous psychological pressure, which results in psychological stress. Prolonged exposure to large amounts of stress leads to increased burnout syndrome. The COVID-19 pandemic has led to a significant outflow of medical professionals, leaving the healthcare system unsustainable to work with large numbers of patients [28]. In addition, the pandemic has contributed to additional workload and led to an imbalance between job demand and job resources [15], which is another factor that increases the burnout of healthcare workers [29]. Moreover, some studies showed that stress at work is significantly higher among healthcare professionals working in COVID-19 hospitals than those not working with COVID-19 patients [28]. During the pandemic, research on job satisfaction found that the pandemic did not significantly affect healthcare workers’ job satisfaction but greatly contributed to the workload [30]. However, some studies showed that the job satisfaction of healthcare professionals who contact patients with COVID-19 is lower than that of those who do not [31].

Due to the aging population, there is a growing need for physiotherapists, and more and more schools and faculties for physiotherapists are opening in Croatia. In the past five years, the number of enrolled physiotherapy students has increased by almost 60%. In addition, many physiotherapists are confronted with different ways of working and approaches to working with individual patients. There are data on physiotherapists’ burnout ranging from 15.7% in Italian physiotherapists [18], over 22.5% in Poland [11], to 42% in Portuguese physiotherapists [23]. However, there are no data on occupational burnout and job satisfaction in Croatian physiotherapists. Therefore, this study aimed to determine Croatian physiotherapists’ job satisfaction and occupational burnout and explore the association between sociodemographic variables, job satisfaction, and occupational burnout.

## 2. Participants and Methods

### 2.1. Participants

A cross-sectional study was conducted among physiotherapists in Croatia between December 2021 and February 2022. Physiotherapists were selected based on an invitation sent through groups on social networks specializing in physiotherapy. All physiotherapists working in Croatia who completed the whole questionnaire were included in the study. Therefore, there were no missing values in the questionnaire. The study consisted of 404 Croatian working physiotherapists (343 female and 61 male respondents). According to the Croatian Institute of Public Health, in 2020, 3446 physiotherapists were employed in Croatia [32]. The power analysis revealed that 346 respondents were needed to achieve a power of 0.95 at an alpha level of 0.05. The Ethical Committee of the Faculty of Dental Medicine and Health Osijek approved the study (No. 2158/97-97-07-21-29), and all participants gave digital informed consent. The study was conducted online according to the Declaration of Helsinki and its amendments.

### 2.2. Instruments

The questionnaire contained three parts. The first part included sociodemographic data such as age, gender, workplace (government vs. private), years of work experience, education level (technician vs. bachelor vs. master), and marital status. The other two parts were related to job satisfaction and burnout.

The Job Descriptive Index (JDI) measures job satisfaction [33]. The JDI has been described as the most popular and most frequently used measure of job satisfaction [34]. The study used an abbreviated version of the JDI to measure job satisfaction, consisting of 25 items that include five facets: satisfaction with coworkers, supervisors, work itself, pay, and promotion opportunities [34]. Before this study, the JDI questionnaire was translated into Croatian according to Sousa and Rojjanasrirat [35]. Furthermore, it was validated in the Croatian population of the teacher [6]. In the present study, Cronbach alphas for the JDI facets were as follows: for coworkers, 0.219; supervisors, 0.697; work itself, 0.726; pay, 0.629; and promotion opportunities, 0.658. The Cronbach alpha in the total sample for the whole JDI scale was 0.907.

The Oldenburg Burnout Inventory (OLBI) was designed in German [36,37] and measures burnout with two dimensions—disengagement and exhaustion. Each dimension consists of eight items. Four are positively worded, and four are negatively worded [38], thus increasing psychometric properties compared to the Maslach Burnout Inventory, which includes only positively worded items [38]. The Maslach Burnout Inventory measures three dimensions of burnout, compared to two in OLBI. The exhaustion in the Maslach Burnout Inventory covers the affective aspects, while OLBI covers additional the physical and cognitive aspects. This simplifies the use of the OLBI for workers who perform physical tasks [38]. In Maslach’s Burnout Inventory, depersonalization refers to the emotional distance from the patient [38]. Disengagement refers to one’s distance from work in general and cynical moods, while exhaustion results from intense physical, affective, and cognitive stress [27,39]. In OLBI, depersonalization is a form of disengagement [37]. There is a more significant similarity between cynicism and disengagement. However, items of cynicism mainly refer to a lack of interest in the job [38]. Both OLBI dimensions were reliable. However, negatively framed items are not highly and linearly related to positively framed items, and it is suggested to use nonparametric analysis methods in studies with OLBI [37]. The OLBI results were obtained by calculating the average of all items in a particular burnout dimension. A higher score indicates greater disengagement and exhaustion [40]. The cut-off values for exhaustion ≥2.25 indicate high exhaustion, while ≥2.10 indicates high disengagement [27]. The present study used the OLBI from a Croatian translation of the OBLI previously used in similar studies [41,42]. In the Croatian OLBI adaptation, Cronbach alphas were 0.84 and 0.76 for disengagement and exhaustion dimensions [41].

These instruments were chosen because they are widely used, and there is strong evidence of their constructive validity and reliability.

### 2.3. Statistical Analyses

Cronbach alphas were determined through factor analysis to evaluate the reliability of the instruments. Due to the results of the Kolmogorov–Smirnov test on the JDI facets and OLBI dimensions, nonparametric statistical tests were used for analyses. Sociodemographic categorical variables were described with absolute and relative frequencies, and numerical sociodemographic variables and variables related to burnout and job satisfaction were presented as mean and standard deviations (SD). The Mann–Whitney and Kruskal–Wallis tests compared these factors against sociodemographic variables. Spearman correlation analyses were used to explore associations between burnout and job satisfaction variables. In addition, multivariate linear regression analyses were conducted to demonstrate the independent effects of sociodemographic variables on OLBI dimensions and individual JDI facets. The *p*-value ≤ 0.05 was deemed statistically significant. All analyses were performed using SPSS software (ver. 22.0, SPSS Inc., Chicago, IL, USA).

## 3. Results

### 3.1. Sociodemografic Data

The present study included 404 Croatian physiotherapists in the statistical analyses. The majority of respondents were female (84.9%), with an average age of 34 ± 9 years, working in a private institution (50.3%), and married (49.3%). The characterization of the physiotherapists is presented in Table 1.

### 3.2. Job Satisfaction Assessed by Job Descriptive Index

Overall scores for the coworkers, supervisor, work itself, pay, and promotions facets of the JDI were calculated by summing the values of the five items for each factor. The range of scores on each scale was from 0 to 25. Higher scores indicated higher job satisfaction. For example, the average JDI scores for the coworkers’ facet was 18.66 ± 2.96, supervisor facet was 16.87 ± 4.21, work itself facet was 17.24 ± 4.38, pay facet was 17.25 ± 3.85, and promotional opportunities facet was 18.1 ± 3.79. Most physiotherapists were satisfied with coworkers, promotional opportunities, pay, and supervisors. However, physiotherapists were less satisfied with the work itself.

The differences between the sociodemographic data of respondents in five JDI facets are shown in Table 2. Female physiotherapists were more satisfied with pay and promotional opportunities than their male counterparts (*p* = 0.03). At the same time, satisfaction with coworkers and supervisors was higher in female but with borderline statistical significance (*p* = 0.06). A significant difference was noted between physiotherapists’ workplaces. Those working in private institutions were more satisfied with their supervisor, pay, and work (*p* < 0.001). In addition, multivariate regression analyses were performed to examine the effects of sociodemographic variables on JDI facets (Supplementary Table S1). There were no statistically significant relationships between age, years of professional experience, education levels, and JDI facets. However, there was a significant relationship between marital status and supervisor and promotion facets of the JDI. Single physiotherapists were less satisfied with supervisors and promotion opportunities.

### 3.3. Burnout Assessed by the Oldenburg Burnout Inventory

The average score of the disengagement dimension of burnout assessed by OLBI was 2.05 ± 0.68, and the exhaustion dimension was 2.23 ± 0.66. The differences between female and male physiotherapists in both dimensions were insignificant. However, physiotherapists working in government institutions expressed higher scores on the disengagement dimension than those working in private institutions (Table 3). Multivariate linear regression analyses confirmed that difference. Moreover, a significant relationship was found between marital status and the disengagement dimension of the OLBI, where married physiotherapists were less disengaged (Supplementary Table S2).

### 3.4. Job Satisfaction and Burnout

Spearman correlation analysis revealed a statistically significant negative correlation between all JDI facets scores and OLBI scores in exhaustion and disengagement dimensions. In contrast, significant positive correlations were observed between the JDI aspects (Table 4).

A multiple regression analysis was performed to examine whether JDI facets significantly predicted burnout scores and vice versa. JDI facets promotion, supervisor and work significantly contributed to disengagement. The promotion was the only factor significantly contributing to the exhaustion dimension of OLBI (Table 5).

## 4. Discussion

The present study showed that physiotherapists are satisfied with their job, especially with their coworkers, and opportunities for promotions. In contrast, they are least satisfied with their supervisors. Furthermore, it has been shown that Croatian physiotherapists have a moderately high level of occupational burnout in the dimension of disengagement (2.05 ± 0.68) and exhaustion (2.23 ± 0.66), which are very close to the limit values. However, those scores are below the cut-off values of 2.10 and 2.25 for disengagement and exhaustion. In addition, it has been observed that higher job satisfaction is associated with a lower risk of physiotherapist occupational burnout.

This study results are in line with the results of previous research [4,12]. Croatian physiotherapists are particularly satisfied with their coworkers. Although the overall results indicate a somewhat high level of occupational burnout of physiotherapists on both dimensions, disengagement and exhaustion, physiotherapists working in government institutions are more significantly exposed to high levels of disengagement, which is consistent with similar research [11].

Job satisfaction is affected by several demographic variables. Thus, female physiotherapists are more satisfied with each of the five facets of the JDI scale than their male counterparts, which agrees with previous research [9,43]. A significant difference was confirmed in the greater satisfaction of women with pay and opportunities for promotions than in men. Perceived higher levels of job satisfaction among women may result from different expectations and priorities regarding their work than men [9]. Physiotherapists employed in private institutions are more satisfied with their job than physiotherapists employed in government institutions. This difference is significant in the facets of supervisors, work itself and pay. Similar results were obtained by Latzke et al. [12] and Salles et al. [14]. They showed that self-employed physiotherapists were more satisfied with their job because they could independently organize work and working hours with better financial compensation. In addition, although research has shown that older physiotherapists are more satisfied with jobs than younger ones [7,43], this study found that younger physiotherapists are more satisfied with supervisors and work itself. At the same time, no significant differences were observed between the other three facets of the JDI scale. Higher satisfaction with certain aspects of the work of younger physiotherapists may be associated with the initial phase of enthusiasm [44]. However, the relationship between age and job satisfaction in different studies is often U-shaped. For example, job satisfaction declined until the mid-30s and gradually increased until the late 60s [43]. The fact that younger physiotherapists are satisfied with their work in this study may be a consequence of them making up 45.5% of respondents compared to 21.3% of physiotherapists older than 41 years. Additionally, the only considerable difference concerning years of professional experience was observed for satisfaction with work itself, while other research has shown that job satisfaction increases with professional experience [11,43,45]. The reason for this difference in the present study may be that the majority of physiotherapists (45.5%) are younger than 30 years old and have less than ten years of professional experience (61.4%), while in the study by Sliwinski et al., 31.5% of physiotherapists were under the age of 30 and 46% had professional experience of fewer than ten years [11]. The level of education did not significantly affect job satisfaction in this study. Although some research suggests that the level of education is an important factor in job satisfaction, McIntyre et al. showed that higher education is associated with higher job satisfaction, pay, promotion opportunities, and supervisors [43]. However, other research shows that more educated physiotherapists have higher pay, more diverse jobs, and more demanding jobs associated with increased stress and reduced job satisfaction [46]. Therefore, education alone is not crucial to physiotherapists’ job satisfaction, which is confirmed by the results of this study, where no association was found between the level of education and job satisfaction. In addition, marital status also affects job satisfaction. Married physiotherapists and those in a relationship are more satisfied with work itself, supervisors, and opportunities for promotions than single physiotherapists. It has been shown that single people have a higher stress level at work than married healthcare professionals and are less satisfied with their job [47]. Talking to loved ones relieves stress, gives people resilience and increases job satisfaction [48].

Physiotherapists belong to a group of occupations with an increased risk of occupational stress. In addition to affecting the physiotherapist’s job satisfaction, demographic variables also affect their burnout at work. Numerous studies on healthcare professionals observed a higher burnout rate in women than in men [17,20,21]. However, this trend has not been observed in the study on physiotherapists in Croatia. Although men had a slightly higher level of disengagement in this study, women who had a higher level of exhaustion did not significantly differ. Although physiotherapy requires some physical strength, many musculoskeletal disorders can affect job satisfaction and lead to occupational burnout. This is particularly important regarding gender differences because anthropometric characteristics are often associated with musculoskeletal disorders, which are more common in women than men [24]. However, this research showed that women are statistically more satisfied with a job than men. At the same time, there are no differences in burnout levels between women and men, which indicates that physical strength alone does not significantly affect job satisfaction and the performance of physiotherapists. Significant differences in disengagement levels have been observed between the private and government institution, especially in the disengagement dimension, which is in line with other research [38,39]. The Croatian government institutions offer lifelong job security. In contrast, private institutions provide more flexibility, better financial benefits, and opportunities for promotions, but at the same time, physiotherapists are exposed to greater demands at work [38]. The level of occupational burnout did not change with age or years of professional experience in the present study, unlike other studies showing that the physiotherapist’s length of professional experience is associated with a higher risk of burnout [25]. It is known that marital status affects burnout and that single people have higher levels of burnout than married people [49,50]. Thus, in this study, it was observed that single physiotherapists have a higher level of disengagement and exhaustion than married physiotherapists or physiotherapists in a relationship. In addition, a significant difference was observed in the disengagement dimension, which is associated with physiotherapist depersonalization that leads to physiotherapists’ indifferent attitude toward patients. The family environment and partner provide security and support and protect the physiotherapist from developing cynical and negative attitudes toward colleagues in the workplace. Research has shown a transparent link between burnout and education levels. Usually, higher occupational burnout occurs in people with higher education [3,51,52]. Although no significant differences in occupational burnout were observed in this study concerning the level of education, it is evident that physiotherapy technicians have the highest levels of exhaustion (Table 3). Less-educated physiotherapists probably have a reduced ability to apply coping strategies and are exposed to high job demands, thus reducing their sense of personal achievement [53]. However, it is assumed that higher-educated and more-experienced physiotherapists are employed in more responsible and stressful positions, leading to exhaustion and higher levels of occupational burnout [54]. However, this study has not confirmed this because most physiotherapists in Croatia perform similar jobs, regardless of their level of education.

Research conducted in various medical professions, from nurses to physicians, has indicated a significant relationship between job satisfaction and occupational burnout. Furthermore, the lower the level of exhaustion, the higher the job satisfaction [55,56,57]. Higher burnout scores are associated with lower job satisfaction ratings [39]. Increased job satisfaction is strongly associated with pay, interest in the job, and opportunities for promotions [5]. These results are similar to the results presented in this study, where job satisfaction is negatively associated with the disengagement and exhaustion of physiotherapists. Data from this study contribute to the association of physiotherapists’ occupational burnout and job satisfaction with more evidence, as it determines the correlation of burnout syndrome. In addition to the aforementioned findings, the impact of the COVID-19 pandemic in which the research was conducted is certainly not negligible. Studies have shown that healthcare professionals who have been in immediate contact with COVID-19 patients have been exposed to much higher stress and increased levels of occupational burnout than those who have not [28]. However, the job-satisfaction study results during the pandemic are contradictory. While some studies claim that healthcare workers were less satisfied with their job during the pandemic [31], others did not find any differences [30]. These results should be taken with caution because such studies were mostly carried out on healthcare professionals in immediate contact with COVID-19 patients, such as nurses and physicians, while physiotherapists were generally not in such situations. Furthermore, physiotherapists mainly deal with the rehabilitation of post-COVID-19 patients and are therefore exposed to secondary traumatic stress [15].

This study has some limitations. First, the study was conducted as cross-sectional: it can only highlight observations and possible connections, but it cannot show causality. Furthermore, the study was performed using a self-assessment questionnaire whose answers may be affected by the bias of the physiotherapist. In addition, the sample may be biased because it is possible that only those physiotherapists who are either very satisfied or dissatisfied with their job participated in the study. Furthermore, as women are generally more represented in healthcare professions, such as physiotherapists, the gender distribution in the study sample illustrated the overall gender distribution among all physiotherapists in Croatia. This is in accordance with the data from the Croatian Health Statistics Yearbook 2020, which shows that there are 23.8% male physiotherapists in Croatia [32].

## 5. Conclusions

The present study showed that Croatian physiotherapists are satisfied with their job and have a moderately high level of occupational burnout. Factors such as age, gender, work experience, and marital status have been recognized as the main determinants of job satisfaction. Pay is a significant indicator of job satisfaction. The frequency of burnout among physiotherapists is higher among employees in government institutions than in public institutions and among single people compared to those who are married or in a partnership. Exhaustion is the most apparent manifestation of occupational burnout that positively correlates with the workload. It is crucial to monitor and promote the job satisfaction of physiotherapists to reduce the negative impact on medical-care recipients. Therefore, assessing the factors influencing job satisfaction and burnout in the workplace can help develop physiotherapists’ mental health prevention strategies.

## Figures and Tables

**Table 1:** Characteristics of the study group (N = 404).

Features	Mean	SD
Age (years)	34	9
Years of work experience	10.42	9.53
Gender	N	%
Male	61	15.1
Female	343	84.9
Education		
Physiotherapy technician	53	13.1
BSc in physiotherapy	239	59.2
MSc in in physiotherapy	112	27.7
Workplace		
Government institution	201	49.8
Private institution	203	50.3
Marital status		
Married	199	49.3
Partnership	104	25.7
Single	101	25

SD—standard deviation.

**Table 2:** The Job Descriptive Index facets results in the physiotherapists’ study group and selected subgroups (N = 404).

Facets			Coworkers	Supervisor	Work Itself	Pay	Promotions
			Mean ± SD	Mean ± SD	Mean ± SD	Mean ± SD	Mean ± SD
	**Total**	(*N* = 404)	18.66 ± 2.96	16.87 ± 4.21	17.24 ± 4.38	17.25 ± 3.85	18.15 ± 3.79
Gender	Male	(*n* = 61)	17.92 ± 3.16	15.87 ± 4.47	16.23 ± 3.85	16.33 ± 3.85	17.13 ± 4.04
	Female	(*n* = 343)	18.79 ± 2.90	17.04 ± 4.14	17.42 ± 4.32	17.41 ± 3.83	18.33 ± 3.72
		Z	−1.86	−1.91	−1.68	−2.12	−2.08
		*p* *	0.06	0.06	0.09	**0.03**	**0.03**
Workplace	Government institution	(*n* = 201)	18.61 ± 2.93	15.99 ± 2.93	16.05 ± 4.09	16.50 ± 3.56	17.95 ± 3.67
	Private institution	(*n* = 203)	18.71 ± 2.99	17.73 ± 4.21	18.42 ± 4.33	17.98 ± 3.98	18.35 ± 3.90
		Z	−0.78	−4.17	−5.64	−4.22	−1.26
		*p* *	0.44	**<0.001**	**<0.001**	**<0.001**	0.21
Education	Physiotherapy technician	(*n* = 53)	18.17 ± 3.16	16.21 ± 4.12	16.15 ± 3.95	17.08 ± 3.46	17.42 ± 3.34
	BSc in physiotherapy	(*n* = 239)	18.77 ± 2.89	17.14 ± 4.03	17.51 ± 4.27	17.36 ± 3.69	18.33 ± 3.82
	MSc in in physiotherapy	(*n* = 112)	18.67 ± 2.99	16.61 ± 4.57	17.19 ± 4.73	17.08 ± 4.35	18.11 ± 3.89
		H	1.42	2.71	4.33	0.76	2.92
		*p* **	0.49	0.26	0.12	0.69	0.23
Age	19–30 years old	(*n* = 184)	18.81 ± 2.85	17.50 ± 4.11	17.89 ± 4.53	17.55 ± 3.84	18.35 ± 3.84
	31–40 years old	(*n* = 134)	18.72 ± 2.88	16.60 ± 4.10	17.08 ± 4.17	17.13 ± 3.82	18.25 ± 3.71
	41–64 years old	(*n* = 86)	18.26 ± 3.27	15.92 ± 4.39	16.10 ± 4.14	16.78 ± 3.89	17.56 ± 3.79
		H	1.95	9.8	10.54	2.95	3.07
		*p* **	0.38	**0.01**	**0.01**	0.23	0.22
Years of professional experience	1–10 years	(*n* = 248)	18.69 ± 2.97	17.16 ± 4.26	17.61 ± 4.56	17.41 ± 3.94	18.19 ± 3.94
	11–20 years	(*n* = 98)	18.60 ± 2.93	16.76 ± 4.09	16.98 ± 3.95	17.08 ± 3.72	18.39 ± 3.35
	over 21 years	(*n* = 58)	16.64 ± 2.98	15.81 ± 4.05	16.10 ± 4.09	16.81 ± 3.69	17.53 ± 3.84
		H	0.27	5.73	6.77	1.89	2.04
		*p* **	0.87	0.06	**0.03**	0.39	0.36
Marital status	Married	(*n* = 199)	18.83 ± 2.86	16.98 ± 4.27	17.13 ± 4.32	17.39 ± 3.84	18.29 ± 3.81
	Partnership	(*n* = 104)	18.64 ± 3.03	17.32 ± 4.04	18.09 ± 4.23	17.61 ± 3.94	18.64 ± 3.76
	Single	(*n* = 101)	18.34 ± 3.07	16.17 ± 4.20	16.59 ± 4.54	16.59 ± 3.73	17.36 ± 3.68
		H	2.10	4.03	6.71	4.58	6.23
		*p* **	0.35	0.13	**0.04**	0.10	**0.04**

SD—standard deviation; * Mann–Whitney test; ** Kruskal–Wallis test. The bold are statistically significant values.

**Table 3:** The Oldenburg Burnout Inventory dimensions result in the study group of physiotherapists and selected subgroups.

Dimensions		Disengagement	Exhaustion
		Mean ± SD	Mean ± SD
	All	2.05 ± 0.68	2.23 ± 0.66
Gender	Male	2.17 ± 0.63	2.21 ± 0.63
	Female	2.02 ± 0.69	2.23 ± 0.66
	Z	−1.81	−0.15
	*p* *	0.07	0.88
Workplace	Government institution	2.11 ± 0.69	2.25 ± 0.65
	Private institution	1.98 ± 0.67	2.21 ± 0.66
	Z	−1.95	−0.85
	*p* *	**0.05**	0.39
Education	Physiotherapy technician	2.04 ± 0.65	3.41 ± 0.67
	BSc in physiotherapy	2.04 ± 0.68	2.19 ± 0.63
	MSc in in physiotherapy	2.07 ± 0.71	2.22 ± 0.69
	H	0.13	4.78
	*p* **	0.94	0.09
Age	19–30 years old	2.04 ± 0.67	2.18 ± 0.62
	31–40 years old	2.06 ± 0.70	2.21 ± 0.67
	41–64 years old	2.04 ± 0.68	2.36 ± 0.69
	H	0.06	4.34
	*p* **	0.97	0.11
Years of professional experience	1–10 years	2.03 ± 0.69	2.20 ± 0.65
	11–20 years	2.08 ± 0.68	2.21 ± 0.64
	over 21 years	2.04 ± 0.68	2.37 ± 0.73
	H	0.53	3.12
	*p* **	0.77	0.21
Marital status	Married	2.01 ± 0.67	2.23 ± 0.69
	Partnership	1.90 ± 0.63	2.19 ± 0.59
	Single	2.26 ± 0.70	2.27 ± 0.64
	H	15.02	0.78
	*p* **	**0.001**	0.68

SD—standard deviation; * Mann–Whitney test; ** Kruskal–Wallis test. The bold indicates statistically significant values.

**Table 4:** The Spearman correlations between JDI and OLBI results (N = 404).

	Supervisor	Work Itself	Pay	Promotions	Disengagement	Exhaustion
JDI–Coworkers	0.590 **	0.506 **	0.574 **	0.688 **	−0.400 **	−0.330 **
JDI–Supervisor		0.812 **	0.820 **	0.764 **	−0.441 **	−0.337 **
JDI–Work itself			0.828 **	0.722 **	−0.563 **	−0.374 **
JDI–Pay				0.730 **	−0.511 **	−0.341 **
JDI–Promotions					−0.565 **	−0.499 **
OLBI–Disengagement						0.700 **

JDI—Job Descriptive Index; OLBI—Oldenburg Burnout Inventory; ** *p* < 0.01.

**Table 5:** Multivariate regression analyses on Job Descriptive Index facets and Oldenburg Burnout Inventory dimensions (N = 404).

JDI Facets	B	SE	*t*	95% CI	*p*
Coworkers					
Disengagement	−0.21	0.03	−6.16	(−0.28)–(−0.14)	**<0.001**
Exhaustion	−0.02	0.04	−0.64	(−0.09)–0.05	0.52
**Supervisor**					
Disengagement	−0.34	0.05	−7.06	−(0.43)–(−0.24)	**<0.001**
Exhaustion	−0.02	0.05	−0.45	(−0.12)–0.08	0.65
**Work itself**					
Disengagement	−0.49	0.05	−10.97	(−0.59)–(−0.41)	**<0.001**
Exhaustion	0.04	0.05	0.94	(−0.05)–0.14	0.35
**Pay**					
Disengagement	−0.41	0.04	−9.92	(−0.49)–(−0.33)	**<0.001**
Exhaustion	0.05	0.04	1.21	(−0.03)–0.14	0.23
**Promotions**					
Disengagement	−0.34	0.04	−8.99	(−0.42)–(−0.27)	**<0.001**
Exhaustion	−0.11	0.04	−2.86	(−0.19)–(−0.04)	**0.004**

B—unstandardized beta coefficient; SE—standard error; CI—confidence interval; JDI—Job Descriptive Index facets; workplace: 1 = government, 2 = private; gender: 1 = male, 2 = female; education: 1 = technician, 2 = BSc, 3 = MSc; marital status: 1 = married, 2 = relationship; 3 = single. The bold indicates statistically significant values.

## Data Availability

The dataset generated during the study is available from the corresponding author on reasonable request.

## Supplementary Materials

The following supporting information can be downloaded at: https://www.mdpi.com/article/10.3390/healthcare10050905/s1, Table S1: Multivariate regression analyses on Job Descriptive Index facets and sociodemographic data (N = 404); Table S2: Multivariate regression analyses on Oldenburg Burnout Inventory dimensions and sociodemographic data (N = 404).
