# Supplementary material for: Job Satisfaction and Burnout in Croatian Physiotherapists
Source: Healthcare (Basel). 2022 May 13;10(5):905. doi: 10.3390/healthcare10050905 (PMC9140399; doi:10.3390/healthcare10050905)
Supplement: Supplementary file 1 [file healthcare-10-00905-s001.zip › healthcare-1690085-supplementary.pdf]

Supplementary materials

Table S1. Multivariate regression analyses on Job Descriptive Index facets and sociodemographic data (N = 404).

Facets	B	SE	t	95% CI	*p*
**Coworkers**					
Age	-0.06	0.06	-1.08	(-0.17)-0.05	0.28
Workplace	0.09	0.30	0.29	(-0.51)-0.69	0.77
Gender	0.84	0.41	2.03	0.03-1.65	**0.04**
Years of professional experience	0.04	0.05	0.80	(-0.06)-0.14	0.42
Education	0.26	0.24	1.09	(-0.21)-0.74	0.28
Marital status	-0.31	0.19	-1.61	(-0.69)-0.07	0.11
**Supervisors**					
Age	-0.12	0.08	-1.59	(-0.28)-0.03	0.11
Workplace	1.62	0.42	3.86	0.79-2.45	**<0.001**
Gender	1.08	0.57	1.89	(-0.04)-2.21	0.06
Years of professional experience	0.07	0.07	0.92	(-0.08)-0.21	0.36
Education	0.27	0.34	0.79	(-0.39)-0.93	0.43
Marital status	-0.63	0.27	-2.34	(-1.17)-(-0.10)	**0.02**
**Work itself**					
Age	-0.10	0.08	-1.27	(-0.26)-0.06	0.21
Workplace	2.31	0.43	5.35	1.46-3.15	**<0.001**
Gender	1.15	0.59	1.96	(-0.01)-2.29	**0.05**
Years of professional experience	0.05	0.07	0.73	(-0.09)-0.19	0.46
Education	0.58	0.34	1.68	(-0.09)-1.26	0.09
Marital status	-0.45	0.28	-1.61	(-0.99)-0.09	0.11
**Pay**					
Age	-0.04	0.07	-0.61	(-0.19)-0.09	0.54
Workplace	1.47	0.39	0.19	0.71-2.23	**<0.001**
Gender	0.98	0.53	0.09	(-0.05)-2.02	0.06
Years of professional experience	0.02	0.07	0.06	(-0.011)-0.15	0.71
Education	0.12	0.31	0.02	(-0.49)-0.73	0.69
Marital status	-0.46	0.25	-0.09	(-0.95)-0.03	0.07
**Promotions**					
Age	-0.06	0.07	-0.78	(-0.19)-0.09	0.44
Workplace	0.36	0.39	0.94	(-0.39)-1.12	0.35
Gender	0.07	0.53	2.15	0.09-2.17	**0.03**
Years of professional experience	0.31	0.07	0.32	(-0.11)-0.15	0.75
Education	0.33	0.31	1.08	(-0.27)-0.94	0.28
Marital status	-0.53	0.25	-2.13	(-1.02)-(-0.04)	**0.03**

B – unstandardized beta coefficient; SE—standard error; CI—confidence interval; workplace: 1=government, 2=private; gender: 1=male, 2=female; education: 1=technician, 2=BSc, 3=MSc; marital status: 1=married, 2=relationship; 3=single. The bold indicates statistically significant values.

Table S2. Multivariate regression analyses on Oldenburg Burnout Inventory dimensions and sociodemographic data (N = 404).

	**B**	**SE**	**t**	**95% CI**	***p***
**Disengagement**					
Age	0.04	0.10	0.43	(-0.16)-0.25	0.67
Workplace	-1.12	0.56	-2.02	(-2.22)-(-0.03)	**0.04**
Gender	-0.99	0.76	-1.30	(-2.47)-0.50	0.19
Years of professional experience	-0.04	0.09	-0.47	(-0.23)-0.14	0.64
Education	-0.09	0.45	-0.20	(-0.96)-0.79	0.84
Marital status	0.82	0.36	2.28	0.11-1.52	**0.02**
**Exhaustion**					
Age	0.05	0.1	0.48	(-0.15)-0.25	0.63
Workplace	-0.19	0.54	-0.36	(-1.26)-0.87	0.72
Gender	0.16	0.74	0.22	(-1.28)-1.61	0.82
Years of professional experience	0.004	0.09	0.05	(-0.18)-0.19	0.96
Education	-0.56	0.43	-1.29	(-1.41)-0.29	0.19
Marital status	0.41	0.35	1.17	(-0.28)-1.09	0.24

B – unstandardized beta coefficient; SE—standard error; CI—confidence interval; workplace: 1=government, 2=private; gender: 1=male, 2=female; education: 1=technician, 2=BSc, 3=MSc; marital status: 1=married, 2=relationship; 3=single. The bold indicates statistically significant values.
